# Seeking American Society of Clinical Oncology‐Quality Oncology Practice Initiative (ASCO‐QOPI) certification in a northern New England rural health system and cancer care network

**DOI:** 10.1002/lrh2.10415

**Published:** 2024-03-28

**Authors:** Hilary M. Perrey, Evelyn Taylor, Brett F. Cropp, Meaghan J. Bumpus, Shannon Lessard, Jeanette A. Pretorius, Jonathan H. Angus, Megan F. Duperreault, Amanda Snow, Dorothy Wang, Meredith Curtis, Lauren A. Couture, David R. Adolphson, Kimberly Smith, Joy H. Moody, Michael J. Bianchi, Mark G. Parker, Amit Sanyal, Scot C. Remick

**Affiliations:** ^1^ Departments of Information Technology, Medical Education, Medicine, Nursing and Pharmacy, MaineHealth Performance Improvement Team MaineHealth, MaineHealth Cancer Care Network, and Maine Medical Center Portland Maine USA; ^2^ Harold Alfond Center for Cancer Care at Maine General Medical Center Augusta Maine USA; ^3^ Department of Medicine Tufts University School of Medicine Boston Massachusetts USA; ^4^ ASCO Members Alexandria Virginia USA

**Keywords:** accreditation, ASCO‐QOPI, cancer network, oncology, quality improvement

## Abstract

In 2006 following several years of preliminary study, the American Society of Clinical Oncology (ASCO) launched the Quality Oncology Practice Initiative (QOPI). This cancer‐focused quality initiative evolved considerably over the next decade‐and‐a‐half and is expanding globally. QOPI is undoubtedly the leading standard‐bearer for quality cancer care and contemporary medical oncology practice. The program garners attention and respect among federal programs, private insurers, and medical oncology practices across the nation. The MaineHealth Cancer Care Network (MHCCN) has undergone expansive growth since 2017. The network provides cancer care to more than 70% of the cases in Maine in a largely rural health system in Northern New England. In fall 2020, the MHCCN QOPI project leadership, following collaborative discussions with the ASCO‐QOPI team, elected to proceed with a health system–cancer network‐wide QOPI certification. Key themes emerged over the course of our two‐year journey including: (1) Developing a highly interprofessional team committed to the project; (2) Capitalizing on a single electronic medical record for data transmission to CancerLinQ; (3) Prior experience, especially policy development, in other cancer‐focused accreditation programs across the network; and (4) Building consensus through quarterly stakeholder meetings and awarding Continuing Medical Education (CME) and American Board of Medical Specialists (ABMS) Maintenance of Certification (MOC) credits to oncologists. All participants demonstrated a genuine spirit to work together to achieve certification. We report our successful journey seeking ASCO‐QOPI certification across our network, which to our knowledge is the *first‐of‐its‐kind* endeavor.

## 1 INTRODUCTION

**FIGURE 1:**
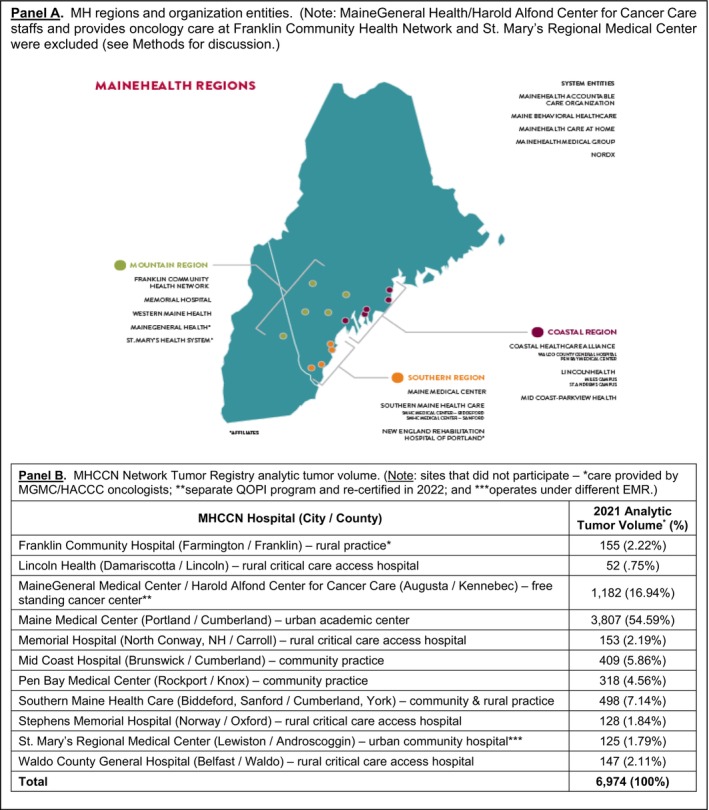
MaineHealth's (MH) three regions with member/affiliate organizations (Panel A); and MaineHealth Cancer Care Network (MHCCN) analytic tumor volume by member site (Panel B).

In June 2017, MaineHealth (MH), a leading integrated health system in the nation announced a $10 million, 5‐year grant from the Harold Alfond Foundation supporting *The Development of an Integrated Patient‐Centered Oncology Service Line for Maine*. The award formally launched the MaineHealth Cancer Care Network (MHCCN), which is comprised of nine MH member organizations and two affiliates at MaineGeneral Medical Center–Harold Alfond Center for Cancer Care in Augusta and St. Mary's Regional Medical Center in Lewiston. In 2023, MH was divided into three regions—Southern, Coastal, and Mountain (Figure 1/Panel A). MaineHealth provides healthcare services to 1.1 million people in 11 of 16 counties in south‐central Maine and Carroll County in eastern New Hampshire. Since December 2018, the MHCCN has undergone expansive growth substantiated by rapid evolution of a network‐wide employed medical oncology group. In August 2019, MHCCN was competitively awarded entry into the NCI Community Oncology Research Program. The Portland practice in October 2020, assumed a major teaching role in the Maine Track Program—Tufts University School of Medicine undergraduate medical education for the second‐year hematology‐oncology course (last 3 years taught in Maine); and in July 2023, launched a 3‐year combined Hematology‐Oncology Fellowship focusing on rural cancer care.

Maine is a small state (population of 1.38 million) and regarded the most rural in the nation based on Rural‐Urban Community Area code criteria.^1^, ^2^, ^3^ It is also the oldest state (median age 44.9 years).^4^ The current age‐adjusted cancer incidence and mortality rates exceed US averages of 478.5 v. 439.8 per 100 000 and 163.7 v. 147.3, respectively.^5^ This corresponds to the 10th and 11th highest incidence and mortality rate(s) in the nation.^5^ In 2019, 9600 cancer cases were diagnosed in Maine.^6^ In 2021, the MHCCN reported an analytic tumor volume of 6974 cases (Figure 1/Panel B), which represents 73% of Maine's cancer burden.^7^

In 1999, the Institute of Medicine published a report that outlined opportunities and potential strategies to improve evidence‐based cancer care nationally.^8^ This report preceded ASCO's efforts to define, measure, and implement a quality‐based cancer program—Quality Oncology Practice Initiative (QOPI).^9^ Between 2003 and 2006, ASCO led a pilot program in three distinct phases involving 23 oncology practices that laid the foundation for the formative ASCO‐QOPI certification.^10^, ^11^ Since 2006, QOPI is now available to practices of any US‐based ASCO member.^10^ Refinements include developing standards based on the ASCO/Oncology Nursing Society for safe chemotherapy administration, oral oncolytic drug standards, and on‐site survey(s).^12^, ^13^ Recently, ASCO‐QOPI standards have been promoted and developed globally.^14^

In fall 2020, we embarked on a feasibility discussion with ASCO seeking a health system–rural cancer network‐wide QOPI certification. We *hypothesize* that pursuit of QOPI certification will have salutary benefits across a rural cancer care network. Illustrative benefits are not limited to establishing quality cancer care across all our practices, once fully operational it will enable network practice leadership to identify quality trends at both the practice and individual provider levels, reduce care variation, and importantly provide documentation of quality cancer care across our network and share with third‐party payers, many of whom identify and/or use QOPI metrics as *state‐of‐the‐art* for contemporary cancer care. We report our experience spanning more than 2 years seeking accreditation across eight member practices. These included an academic practice, three smaller community practices, and four practices at critical care access hospitals providing cancer care in six rural locations (Figure 1/Panel B) in Northern New England.

## 2 METHODS

### 2.1 Background

By 2016, all 11 MHCCN hospitals were integrated into a centralized Network Tumor Registry, which provided an excellent departure point for reporting numerous quality cancer measures. MaineGeneral successfully renewed their QOPI certification in 2022. Their oncologists provide cancer care for patients at Franklin Memorial Hospital in Farmington and accordingly measurement data are not reported from this MH member practice. This arrangement is geographically expedient for rural cancer care in our health system and has been operational for 7 years. The Franklin practice, however, follows all MHCCN (QOPI) policies. MaineGeneral provided valuable mentorship to the MHCCN team over this journey. By January 2021, all MH member oncology practices operated under a single electronic medical record (EMR)—*Epic Systems* (Verona, WI). This arrangement facilitated the decision to electronically report measurement data to CancerLinQ.^15^, ^16^ St. Mary's Regional Medical Center utilizes a separate instance of Epic hosted on their premises and managed by their organization. The MHCCN team does not have access to data within St. Mary's Epic instance. Given this, including St. Mary's Epic data would have required a separate Business Associate Agreement (BAA), dedicated IT resources, and an inventory of their workflows and Epic configuration items. We elected to defer including St. Mary's for these reasons. In collaboration with the ASCO‐QOPI administrative team, mutually agreeable accommodations for these three sites were made. With this backdrop, the MHCCN team reviewed published ASCO‐QOPI Standards Manual and QOPI Certification Track 2021 Measures Summary (Table 1).^17^, ^18^

**TABLE 1:** Quality Oncology Practice Initiative certification measures and policies.

SmartLinQ QOPI® Certification Track 2021 Measures Summary for electronic reporting		
Module	Measure	Measure title
Core	2	Staging documented within 1 month of first office visit.
Core	4a	Pain quantification score during first two encounters.
Core	10	Chemotherapy intent (curative vs. non‐curative) documented before or within 2 weeks after administration.
Core	21aa	Tobacco use assessment.
Core	25b	Height, weight, and BSA documented prior to curative chemotherapy.
Core	N/A	Documentation of current medications in the medical record.
Core	QOPI 15	G‐CSF administered to patients who received chemotherapy for metastatic cancer (lower score—better).
Core	QOPI 5	Chemotherapy administered to patients with metastatic solid tumor with performance status of ECOG 3 or 4; KPS 10‐40; or undocumented.
EOL	N/A	Care plan.
EOL	N/A	Proportion receiving chemotherapy in the last 14 days of life.
SMT	27	Corticosteroids and serotonin antagonist prescribed or administered with moderate or high emetic risk chemotherapy.
SMT	28/28a	NKI receptor antagonist and olanzapine prescribed or administered with high emetic risk chemotherapy.
BR	54	Her‐2/neu testing for breast cancer patients.
BR	59	Hormonal therapy for breast cancer patients within 1 year of diagnosis.
BR	QOPI 11	Combination chemotherapy received for breast cancer.
CRC	68	Adjuvant chemotherapy received within 4 months of diagnosis by patients with stage III colon cancer.
GynOnc	94	Platin and/or taxane administered within 42 days of staging for ovarian, fallopian tube, or peritoneal cancer.
Lung	84	Percentage of patients with initial AJCC stage IV or distant metastatic lung cancer whose performance status is documented.
MM	N/A	Treatment with bisphosphonates.
NHL	78a	Hepatitis B testing prior to rituximab administration for NHL.

QOPI policies		
Standard	QOPI policy requirement(s) [MH/MHCCN Supporting Policy]	Ref. nos.
1.1	Clinical staff qualifications of those who order, prepare, document, and administer chemotherapy [MH Validation of Initial Competency and Ongoing Assessment of Oncology Nurses Involved in the Care of Patients Receiving Chemotherapy and Immunotherapy Treatment Policy (Appendix XX. Pharmacy Hazardous Drug Compounding Education and Training Requirements); Advanced Practice Professional (APP) Competencies; Pharmacy Compounding Competencies]	19, 20, 21, 22, 23, 24, 25, 26, 27, 28, 29, 30
1.6	Identifies a process to provide 24/7 triage [MH Medical Oncology Coverage Policy]	──
2.1	The process for obtaining informed consent [MH Informed Consent for Chemotherapy/Biotherapy for Adult and Pediatric Patients Policy]	21, 31, 32
3.5	A policy specifying how intrathecal medication is maintained [MH Nursing Verification, Administration, and Safe Handling of Chemotherapy Biotherapy Policy]	19, 20, 21, 22, 23, 24
3.10	Defines extravasation management procedures [MH Extravasation Management Guidelines]	33, 34, 35, 36
4.1	A policy for emergent treatment of patients [MH Management of Hypersensitivity/Infusion Reaction(s)]	37, 38, 39, 40
4.2	Initial oral adherence assessment policy [MH Corporate Chemotherapy/Biotherapy Treatment Planning, Ordering, Administration Policy]	21, 41
4.3	Ongoing Oral Adherence Assessment Policy [MH Corporate Chemotherapy/Biotherapy Treatment Planning, Ordering, Administration Policy]	21, 41

Abbreviations: BR, breast cancer; CRC, colorectal cancer; EOL—end‐of‐life; GynOnc, gynecologic oncology; MM, multiple myeloma; N/A, not applicable; NHL non‐hodgkin lymphoma; SMT, symptom/toxicity management.

### 2.2 QOPI pre‐certification Phase I—Preliminary work

**FIGURE 2:**
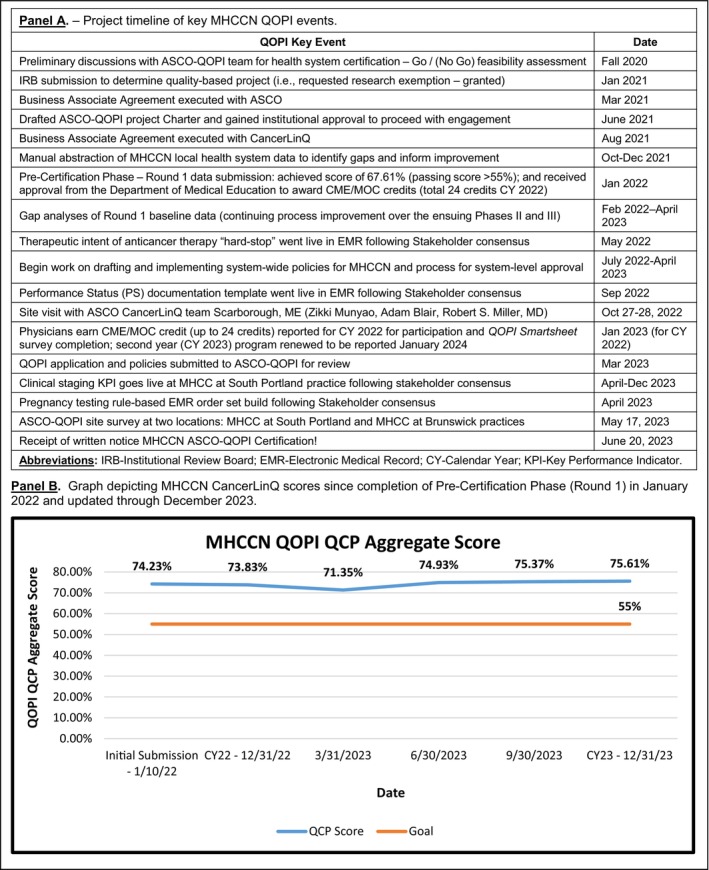
Project timeline (Panel A) and graph depicting MaineHealth Cancer Care Network (MHCCN) CancerLinQ scores over course of project (Panel B).

We initially sought an IRB‐approved research exemption to consider the project quality improvement (Figure 2/Panel A). Thereafter, a BAA and registration to gain access to the Quality Initiative dashboard was executed with ASCO‐QOPI. Our team worked with our Information Technology Department for feasibility assessment for electronic data transfer. This required a project charter and led to another BAA with CancerLinQ. During the onboarding process, CancerLinQ provided the MHCCN team with SQL code to extract data elements they required from Epic. This code targeted Epic's Clarity reporting environment, which is updated nightly with changes from Epic's Hyperspace (front‐end interface). The MHCCN team worked with CancerLinQ to develop code to target the Medical Oncology population from Epic, and to isolate specific Epic configuration items that were needed for measure calculations, including Flowsheets, Note Types, and SmartData Elements. In total, 19 SQL extracts were executed weekly to pull the previous week's data. The MH Integration team established secure delivery to CancerLinQ through the Secure File Transfer Protocol (SFTP). During this period, the team also began to socialize the QOPI initiative across all MHCCN sites and assemble a stakeholder group. Taking advantage of our Network Tumor Registry we embarked upon manual abstracting 2020 QOPI Pre‐Certification measures from up to 10 (randomly selected) charts across representative tumor types including breast, colorectal, and non‐small cell lung cancer cases. This was undertaken at all eight participating member sites to determine baseline assessment, identify gaps, and to help with electronic data transfer (see Table 2). Following review of the manually abstracted data our QOPI team convened once or twice a month via Zoom.

**TABLE 2:** Thirteen common Quality Oncology Practice Initiative measures that we initially recorded as part of our preparatory manual abstraction and carried forward during our electronic transfer.

QOPI measure	Manual abstraction	First electronic submission CY2021	Last electronic submission CY2023
	9/8/2021	1/10/22	12/31/23
Chemotherapy intent documented	98%	71.4%	91.5%
Current medications documented	100%	98.1%	97.9%
Height, weight, BSA documented	100%	99.1%	97.2%
Smoking status/tobacco use documented	100%	99.9%	100%
Her‐2/Neu testing (breast only)	100%	75.1%	66.7%
Corticosteroid and serotonin antagonist administered for moderate/high emetic risk chemotherapy	100%	97.5%	94.1%
Adjuvant chemotherapy received within 4 months for stage III colon cancer	100%	78.9%	69.2%
Advanced care plan discussed/documented	97%	61%	39.2%
Pain intensity quantified by second office visit	53%	90.5%	85.5%
Staging documented within 1 month of first office visit	91%	41.8%	44.5%
G‐CSF administered to patients who receive chemotherapy for stage IV cancer	12%^a^	23%^a^	9%^a^
Chemotherapy administered to patients with metastatic solid tumor with performance status of ECOG 3; KPS 10–40; or undocumented	6.3%^a^	100%^a^	35.2%^a^
Percentage of patients with initial AJCC stage IV or distant metastatic lung cancer whose performance status is documented	100%	27.3%	81.2%

*Note*: The table reports status over time of each metric, while Figure 2, Panel B graphs composite CancerLinQ scores over course of project.

^a^ Denotes lower score is better.

### 2.3 Phases II and III—Electronic data transfer to CancerLinQ

CancerLinQ did not supply Application Programming Interface(s) (APIs) for MHCCN to programmatically extract scores from their environment. MHCCN worked with CancerLinQ to establish the functionality to manually export measure data from their dashboard in a Comma Separated Values (CSV) file. The measure data included numerator, denominator, and actionable patient counts. MHCCN established an Excel template to be able to calculate their QCP score based on CancerLinQ's scoring algorithm, which weighs each measure based on the denominator value. MHCCN also calculated the lowest/highest possible scores for each measure and total based on the number of actionable patients. This method was used to generate the QCP score(s) for the graph in Figure 2/Panel B. In October 2022, a valued component of this phase was a CancerLinQ site visit convened in Scarborough. We shared progress on data reporting and updated CancerLinQ applications for our practices.

### 2.4 Stakeholder meetings

In December 2021, the QOPI leadership team assembled our first stakeholder group meeting. The group is comprised of all MHCCN oncology providers, pharmacists, select Advance Practice Professionals (APPs), and practice managers, and our informatics team is devoted to the project. These forums were convened quarterly, chaired by our leadership team with an agenda distributed in advance, and minutes kept. Physicians were required to complete a post‐meeting survey. A monthly electronic newsletter was distributed that reported progress to date.

### 2.5 Continuing Medical Education/Maintenance of Certification credit for oncologists

Upon receipt of the *SmartLinQ QOPI Certification Registry Submission Report* (Pre‐Certification Phase) in January 2022, we submitted an application to the Department of Medical Education (DME) to offer Continuing Medical Education (CME) and corresponding Maintenance of Certification (MOC) credits to our physicians. We submitted a formal institutional application (*Quality Improvement Intake Form*) since MH is a sponsoring program of the American Board of Medical Specialists (ABMS) Portfolio Program, included our results for each of the 20 QOPI measures, and defended our processes for quality improvement over the ensuing year. Our proposal, titled *FY22 ASCO‐QOPI Clinical Transformation*, was accepted to award a total of 24 credits annually with successful completion of the work. On a monthly basis, 1.5 CME/MOC (subtotal 18) credits were awarded to both acknowledge and reward provider effort to record EMR data in discrete searchable fields. An additional 1.5 CME/MOC credits (subtotal six) were awarded for participation and survey completion following the quarterly stakeholders meeting. These surveys invited feedback on ways forward and developed provider consensus on strategies to build into the EMR to facilitate data capture; thus participants actively contributed to *Plan‐Do‐Study‐Act* cycles. Participation was monitored and recorded quarterly with credits reported to the DME and the American Boards of Internal Medicine and Neurology and Psychiatry (participating neuro‐oncologist). Data were reported to the ABMS for calendar year 2022 in February 2023 and awarded to all participating physicians. To receive credit physicians were required to submit their unique ABMS and individual National Provider Identification (NPI) numbers.

### 2.6 Phase IV – Preparation for QOPI certification and policies development

Project leadership and analyst teams were regularly monitoring progress. Analysts conducted manual reviews of QOPI measure scores and data at the patient/provider level to ensure CancerLinQ accurately portrayed MH performance.

We embarked on formal QOPI policies development during summer 2022 (Table 1). Some policies were already available and periodically updated by the MHCCN Clinical Oncology Practice (COP) Committee (established 2017). This interprofessional committee includes all sites, meets monthly, and charged with developing chemotherapy orders and clinical guidelines (e.g., antiemetic guidance among others). Historically, policies emanating from the COP Committee were primarily for Maine Medical Center but provided to other sites for reference and/or to incorporate in local health system policies. With MH reorganization and our QOPI initiative, an approach to adopt system‐level policies was undertaken. Drafts were developed by the QOPI leadership team and subsequently sent to COP Committee for initial approval; from there nursing policies were routed to the MH Nursing Council and all others to the Clinical Leadership Council chaired by the MH Chief Nursing and Medical Officers respectively for final approval. Once approved all policies were loaded on MCN Policy Manager, an enterprise policy management software (ellucid, MCN Healthcare, Denver, CO). The software icon is located on all desktops for ready reference across the MHCCN.

### 2.7 Phase V—Survey preparation

Leading up to survey the leadership team and stakeholder group convened several ad hoc meetings to review final preparations. We worked closely with the ASCO‐QOPI surveyor conveying policies, answering queries, assisting with organizing the on‐site review, and selecting two sites for survey.

## 3 RESULTS

### 3.1 QOPI pre‐certification Phase I

In January 2022, we achieved an initial Round 1 passing score of 67.61% (target ≥55%) required for the QOPI Pre‐Certification Track. With cumulative reporting of CancerLinq data, our original January 2022 submission score is now 74.23%, which improved to 75.61% as of December 2023 (see Figure 2, Panel B). Our manual abstraction observed nearly 90‐100% compliance on the majority of measures (Table 2). While several measures were documented in the EMR, they were not in discrete “searchable” fields for electronic transfer. Six QOPI measures/standards are worthy of comment (Table 1).

Staging documentation (Core 2) and Performance Status (PS) were nearly uniformly entered in EMR office notes, though not in searchable fields. Our lung cancer PS score (NSCL84) was our lowest at 5.19 (4/77). Chemotherapy intent, curative versus non‐curative (Core 10), while a defined and required element in our informed consent form for treatment, which is scanned into the EMR, is also not searchable. Use of G‐CSF (QOPI15) in patients receiving chemotherapy for metastatic cancer (lower score is better) at 20.95 (44/210) was judged to be slightly higher than it should be. As we approached survey an admitted gap was to develop a consistent approach for pregnancy testing in women of childbearing potential (QOPI Standard 1.2.4), who are about to receive chemotherapy. These issues were addressed at the stakeholder meetings. When using CancerLinQ for automated abstraction, a passing QCP score of ≥55% is required.

Antiemetic therapy for high‐risk antineoplastic agents proved challenging. Our Round 1 score (SMT28/28a) was among the lowest at 12.34 (10/81). This measure requires use of an NK1 receptor antagonist and olanzapine. We reviewed our guidelines and discussed this with our providers and pharmacy teams. First, this was not judged a clinical problem from our patient experience and interdisciplinary discussion among providers, pharmacists, and chemotherapy nurses. Manual abstraction revealed that neither drug was administered nor one of the two agents in the majority of instances. We elected not to aggressively pursue this measure and shared this recommendation with the stakeholder group. Rationale was based on an unsubstantiated problem for our patients, would entail new purchasing arrangements, and potential for polypharmacy and introducing new drug interactions and side effects. Our current practice administers a combination of palonosetron (5‐HT3 antagonist), fosaprepitant (NK1 antagonist), and dexamethasone in this setting with excellent results. We reached out to ASCO‐QOPI to share our concerns about this measure and learned this has been previously raised by other practices. On balance, we thought it prudent to defer managing up on this particular measure.

### 3.2 Phases II/III

The largest barrier to technical implementation proved to be a lack of standardization in documentation across facilities and providers, in addition to limited structured data (e.g., cancer staging). For instance, due to Epic build incompleteness, a range of PS scores were not being transferred into the reporting environment. This significantly contributed to score reductions.

### 3.3 Stakeholder meetings and CME/MOC credits

Participation by providers, other healthcare team members, and our QOPI leadership was robust. Physicians valued participation as well as their input on addressing ways forward. The meetings were free of commercial bias or other interests. We successfully applied for continuation of this activity for 2023 and 2024.

The Stakeholder meetings provided an interprofessional forum to discuss improvement approaches. A fundamental principle when making revisions to EMR workflows was to develop strategies that were efficient, reduced redundancies, and limited the number of “clicks” to help physicians. At the outset, G‐CSF utilization was reviewed in patients receiving chemotherapy for metastatic disease and it was deemed best to permit clinical discretion for use based on acceptable guidelines (e.g., age, comorbidities, myelotoxic risk of regimen among others) rather than prescribe this. The group was also apprised of foregoing major modifications in our approach to antiemetic support for patients receiving highly emetogenic chemotherapy. These recommendations were valued. Accordingly, following physician consensus (post‐meeting survey responses) and working closely with our informatics team, we built a chemotherapy intent “hard‐stop” in our electronic order sets in May 2022; built a PS documentation template in Office Notes in September 2022; and in April 2023 piloted a tumor staging strategy in our South Portland practice to go‐live across the network if efficient. Finally, the group agreed to modify all of our chemotherapy order sets framed in a “rules‐based” manner for women of childbearing potential defined as between 18 and 50 years of age and excluding patients having undergone tubal ligation, hysterectomy, and/or salpingo‐oophorectomy to have pregnancy testing prior to chemotherapy. While this requires more time to build it was the preferred approach and more expedient to avoid a best‐practice advisory in the EMR.

### 3.4 Phases IV/V—Preparation for QOPI certification and survey

Extensive experience with numerous MHCCN member organization on‐site surveys [e.g., Commission on Cancer (CoC), National Accreditation Programs for Breast Cancer and Rectal Cancer, American College of Radiology among others] provided a solid foundation to prepare for the ASCO‐QOPI survey. Our surveyor was a strong advocate and mentor for our leadership team and was actively engaged in advance of our survey.

### 3.5 Immediate quality improvement outcome—Cancer staging

An illustrative example of the impact of our QOPI accreditation immediately followed our pilot staging quality improvement workflow process that is now going live across our network. We are now able to track this work at the individual practice or provider level. Effective October 1, 2023, our Epic TNM staging key performance indicator (KPI) will deploy across all medical oncology sites. The goal is to stage ≥80% of new cancer patients within 31 days post‐consult using the Epic TNM staging form on the problem list. If staging is not completed within 31 days or at all, providers must communicate a reason to the KPI champion and Pareto charts have been created for missed staging reasons. All data are collected and analyzed over time to help us succeed with this goal. Currently, cancer care network practices at Sanford, Brunswick, and South Portland have this KPI active at their sites. In January 2023, Sanford had a staging rate of 25% and averaged 40.8% over the year through December 2023. The corresponding staging rates at Brunswick and South Portland, our largest practices, had initial staging rates of 50% and 65% in January 2023, and end of December 2023 achieved averages of 46.8% and 62.3%, respectively.

## 4 DISCUSSION

Prior to embarking on this effort, our quality program was largely driven by data reporting to the Commission on Cancer/American College of Surgeons accredited Network Tumor Registry. While cancer is a reportable disease and our cancer registry is robust, the data elements largely surround diagnosis, staging, first course of treatment (including surgery, radiation, and/or systemic therapy), and survival. QOPI focuses on these elements in addition to continuous treatment and follow‐up of cancer patients over the arc of the cancer care continuum devoted to contemporary medical oncology practice. The majority of QOPI measures are not captured in our tumor registry database and provides another reason to participate in this program.

To our knowledge, the QOPI accreditation journey reported herein is the *first‐ever* successfully executed across a health system in an essentially rural cancer care network. Several themes emerged from the start. Foremost, this endeavor involved a highly interactive and engaged interprofessional leadership team of oncologists, nurses, pharmacists, informatics analysts, and quality improvement experts over the course of our journey. This approach is of paramount importance. The team was committed to the project, demanded discipline, and met routinely to monitor progress over the two‐year course seeking QOPI certification. Secondly, strong institutional and informatics support to realize this goal was vital. Without the electronic linkages in our health system, the collective effort of single site‐by‐site, manual abstraction of data across a healthcare system is highly impractical. The difference between achieving a pre‐certification score of >75% for manual abstraction versus ≥55% for electronic transfer must be carefully considered. While a lower benchmark for the latter, the time and effort required at the outset for manual abstraction is comparable to expended effort to track and report data electronically. Now that we have operationalized CancerLinQ electronic data reporting enormous efficiencies in time and effort are realized for each successor accreditation period. We feel this is a justifiable and worthwhile approach that spanned more than 2 years. Attention to identifying EMR searchable fields became readily apparent and the need for a strong informatics team to accommodate and build the changes that are required cannot be understated. This was undoubtedly a much more cost‐effective manner in which to proceed and once built, tees up future activities and engagements. Thirdly, prior extensive experience across a variety of cancer‐focused CoC accreditations (especially for tumor registration, breast and rectal cancer) and American College of Radiology for radiation oncology practice proved valuable. Our robust Network Tumor Registry provided strong leadership for this undertaking and readily identified gaps and helped frame ways forward in the EMR. Finally, creating a Stakeholder group and offering CME/MOC credits was prescient and valued by physicians. This venue provided opportunities to define more optimal workflows, especially electronic, and allow physician‐driven input on clinical decision‐making. Offering professional educational and certification credits placed value on physicians' time and participation.

There are opportunities to provide better care to patients and improve documentation. Cancer staging is an illustrative example. Clinical staging presents challenges across the nation and improvements in our EMR documentation were needed, despite frequent documentation in individual office visit notes.^42^ Access to traceable staging data in the EMR facilitates participation in treatment pathways given the complexities and expense of contemporary cancer treatment, and referral of advanced stage patients for palliative care. Our care teams appreciated the need to develop strategies to protect women of childbearing potential and the adopted approach will clearly benefit patients and providers. The efficiencies we are building into our EMR and the power of the CancerLinQ platform will be realized by our practices in the coming years.^43^ This is nicely illustrated by our recent progress on the Epic TNM staging initiative.

The origins of healthcare quality improvement date back nearly 60 years ago.^44^, ^45^, ^46^

Contemporary cancer care is complex and exorbitantly expensive with numerous alternative payment models and value‐based care initiatives. Accordingly, quality measurement in oncology is rapidly advancing.^47^ Current published observations acknowledge considerable stress in healthcare quality improvement.^48^, ^49^, ^50^ What is clear—physicians genuinely want what is best for their patients, sensitivity to federal and private reimbursement approaches are increasingly tethered to quality initiatives, and strategies that embrace physician input and clinical discretion are likely to be more successful.^50^ Given this, ASCO‐QOPI is a physician‐driven, peer‐reviewed process that our organization embraced and for this reason set upon this journey. The MHCCN is better for this achievement.

## 5 CONCLUSIONS

We report a successful two‐year journey to seek ASCO‐QOPI certification in a rural cancer network. Keys to our success included a robust interprofessional team devoted to the work, a single EMR across our health system and participation by our informatics team, extensive experience with other cancer‐focused accreditations, and willingness to embrace and value physician input and clinical discretion capitalizing upon an innovative approach with our Stakeholder group. In the end, this effort was simply the *“right‐thing‐to‐do”* for our patients, their families, and our providers. By sharing our approach we are hopeful this can guide other rural health system cancer care practices and networks.

## AUTHOR CONTRIBUTIONS

**Evelyn Taylor, Hilary Perrey, Brett Cropp, Joy Moody, Michael Bianchi, Mark Parker, Amit Sanyal, and Scot Remick**: Conception and design. **Hilary Perrey, Meaghan Bumpus, and James Reich**: Administrative support. **Brett Cropp, Shannon Lessard, Lauren Couture, Jeanette Pretorius Jonathan Angus, Megan Duperreault, Amanda Snow, Dorothy Wang, Hilary Perrey, and Evelyn Taylor**: Collection and assembly of data. Manuscript: All authors were accountable for all aspects of the work and participated in writing and final approval of the manuscript.

## FUNDING INFORMATION

This work was supported in part by the Harold Alfond Foundation, Portland, ME.

## CONFLICT OF INTEREST STATEMENT

None of the authors of this manuscript have any conflicts to disclose.
